# Impaired immune responses in the lungs of aged mice following influenza infection

**DOI:** 10.1186/1465-9921-10-112

**Published:** 2009-11-18

**Authors:** Franklin R Toapanta, Ted M Ross

**Affiliations:** 1 Center for Vaccine Research, University of Pittsburgh, Pittsburgh, PA, USA; 2 Center for Modeling Pulmonary Immunity, University of Pittsburgh, Pittsburgh, PA, USA; 3 Department of Microbiology and Molecular Genetics, University of Pittsburgh, Pittsburgh, PA, USA; 4 Current address: Center for Vaccine Development, 685 West Baltimore Street, Health Sciences Facility Bldg 1 (HSF-1), University of Maryland School of Medicine, Baltimore, Maryland 21201, USA

## Abstract

**Background:**

Each year, influenza virus infection causes severe morbidity and mortality, particularly in the most susceptible groups including children, the elderly (>65 years-old) and people with chronic respiratory diseases. Among the several factors that contribute to the increased susceptibility in elderly populations are the higher prevalence of chronic diseases (*e.g*. diabetes) and the senescence of the immune system.

**Methods:**

In this study, aged and adult mice were infected with sublethal doses of influenza virus (A/Puerto Rico/8/1934). Differences in weight loss, morbidity, virus titer and the kinetics of lung infiltration with cells of the innate and adaptive immune responses were analyzed. Additionally, the main cytokines and chemokines produced by these cells were also assayed.

**Results:**

Compared to adult mice, aged mice had higher morbidity, lost weight more rapidly, and recovered more slowly from infection. There was a delay in the accumulation of granulocytic cells and conventional dendritic cells (cDCs), but not macrophages in the lungs of aged mice compared to adult animals. The delayed infiltration kinetics of APCs in aged animals correlated with alteration in their activation (CD40 expression), which also correlated with a delayed detection of cytokines and chemokines in lung homogenates. This was associated with retarded lung infiltration by natural killer (NK), CD4^+ ^and CD8^+ ^T-cells. Furthermore, the percentage of activated (CD69+) influenza-specific and IL-2 producer CD8+ T-cells was higher in adult mice compared to aged ones. Additionally, activation (CD69+) of adult B-cells was earlier and correlated with a quicker development of neutralizing antibodies in adult animals.

**Conclusion:**

Overall, alterations in APC priming and activation lead to delayed production of cytokines and chemokines in the lungs that ultimately affected the infiltration of immune cells following influenza infection. This resulted in delayed activation of the adaptive immune response and subsequent delay in clearance of virus and prolonged illness in aged animals. Since the elderly are the fastest growing segment of the population in developed countries, a better understanding of the changes that occur in the immune system during the aging process is a priority for the development of new vaccines and adjuvants to improve the immune responses in this population.

## Introduction

Influenza virus infects a variety of species, including swine, horses, birds, and humans. Hemagglutinin (HA) and neuraminidase (NA) are the important antigenic proteins on the surface of the virus and both undergo two types of antigenic variation: drift and shift. Antigenic drift involves minor changes in these antigens, while shift involves major changes in these molecules that result from replacement of a gene segment(s). New viral variants, due to antigenic drift, emerge constantly and are responsible for yearly epidemics. In contrast, antigenic shifts can produce new virus strains to which most people have no immunity resulting in pandemics. On average, influenza virus infects 5-10% of the global population and results in approximately 500,000 deaths annually. In the United States, influenza virus infections account for 200,000 cases of hospitalizations and 36,000 deaths [1,2]. Among the most susceptible populations are children, pregnant women, the elderly (>65 years) and people with chronic respiratory diseases.

The fastest growing segment of the United States population is individuals over 65 years of age. The elderly have an increased morbidity and mortality to due influenza, as a result of secondary bacterial and viral infections [3]. Several immunological changes occur in the senescent immune system in humans, including impairments in initiation and activation of the immune response and induction and maintenance of immune memory [4,5]. In the case of influenza infections, even though the hospitalization rates for children less than 5 years and adults over 70 years of age are almost identical, individuals older than 70 years have a 35-fold increase in mortality [6,7]. Vaccination can reduce the rates of hospitalization, however protection induced by immunizations is diminished in the elderly compared to the adults as demonstrated by lower antibody titers and higher rates of respiratory illness [8]. Additionally, cell-mediated immune responses to vaccinations are decreased in the elderly [9,10].

Studies with sub-lethal virus infections in aged mice have closely resembled the human situation indicating a delay in virus clearance. This delay was accompanied by a delay and decrease in T-cell responses [4,5,11,12]. Importantly, age-associated changes in the innate response to virus infection, such as production of interferons (IFN) alpha and beta and the activation of innate cells (macrophages, DCs, granulocytes and NK cells), has been poorly explored.

The study presented in this report, globally evaluated the kinetics of activation and infiltration of the primary innate and adaptive immune cell populations in aged and adult mice following influenza virus infection. There were alterations in APCs in aged animals including the up-regulation of activation markers, especially CD40, which correlated with delayed production of IL-12 and several other cytokines including IFN-γ and IL-6 in the lungs of aged animals. Chemokine production was also altered, which also correlated with a delayed infiltration of innate cells and lymphocytes into lungs. Of these populations, the activation of NK and B-cells was altered and the production of neutralizing anti-influenza antibodies was detected 2 days later in aged mice compared to adult mice. Aged mice also had a significantly lower percentage of activated influenza HA-specific CD8+ T-cells. The alterations in the immune response correlated with a delay in virus clearance and slower recovery in aged animals. In summary, all these findings suggest that early alterations in the innate immune system translate into delayed activation of the adaptive immune responses and consequently in prolonged illness. As the elderly population continues to increase, a better understanding of the changes that the immune system undergoes with the aging process is becoming a key factor for the development of new vaccines and adjuvants to improve the immune responses in this segment of the population.

## Materials and methods

### Virus infections and Animals

Female BALB/c mice (Harlan-Sprague, Indianapolis, IN, USA) were infected with the mouse adapted influenza virus, A/Puerto Rico/8/34 (H1N1) (PR8), at 12-16 weeks of age (adult) or 72-76 weeks of age (aged). Mice were anesthetized with a mixture of ketamine and xylazine and intranasally instilled with 50 μl of PBS containing 50-100 pfu of PR8. Following infection, mice were monitored daily for morbidity (weight loss and sickness score) and mortality. Sickness score included evaluation of ruffled fur, hunched back and activity (Table 1). Animals were treated according to the guidelines of the IACUC of the University of Pittsburgh. All the protocols used were approved by the IACUC of the University of Pittsburgh.

**Table 1:** Symptom Score

Score Symptoms	0 each	1 each	2 each	
Ruffled Fur	Absent	Present		

Hunched Back	Absent	Present		

Activity	Normal	Reduced	Severely Reduced	

Mice were evaluated daily and scored for individual symptoms. Ruffled fur (absent = 0; present = 1), hunched back (absent = 0; present = 1) and activity (normal = 0; reduced = 1; severely reduced = 2) were evaluated. The final score was the addition of each individual symptom score (e.g. an animal showing ruffled fur (1), hunched back (1) and reduced activity (1) was scored as 3). The minimum score was 0 for a healthy mouse and 1-4 for illness, depending on severity.

### Tissue harvesting and cell isolation

Lungs were harvested (n = 3 - 6 per time point per experiment) on days 0, 1, 2, 3, 5, 7, 9, 11, 15 and 19 post-infection. In some specific time points, a higher number of animals were used to confirm results for some assays (see legend on each figure for description on time points with higher number of animals). Lungs were exposed by opening the chest cavity and rinsed with cold 1× PBS (4 ml), through the right heart ventricle. The lungs were then removed and forced into suspension in 1× PBS (4 ml) using a cell strainer (70 μm) and a syringe plunger. Tissue samples were then spun down (2500 rpm, 5 min, 4°C) and the supernatants of the lung homogenates collected and stored at -80°C until analysis. The remaining cell pellet was resuspended in 5 ml of red blood cell lysis buffer (ACK buffer) and incubated for 5 min at room temperature (RT). Cells were then washed (2× - 2500 rpm, 5 min, 4°C) with 15 ml of 1× PBS and resuspended in 1 ml 10% RPMI (Mediatech, Manassas, VA, USA). Total number of viable cells was determined by trypan blue exclusion.

### Plaque assay

Lung virus titers were determined by plaque assay using Madin Darby canine kindey (MDCK) cells (ATCC, Manassas, VA, USA). Briefly, MDCK cells were grown in 6 well plates until 90% of the cell monolayer was confluent. Cells were washed twice in DMEM (Mediatech, Manassas, VA, USA). Lung supernatants were gently thawed on ice and 100 μl of the different dilutions (10^1 ^to 10^6^) were plated on MDCK cells and allowed to adsorb for 1 hour at RT. Excess virus was washed away with DMEM (2×) and cells were overlayed with a 2.5 ml of 1:1 mixture of 1.6% agarose, 2× L-15 medium (Cambrex, East Rutherford, NJ, USA) and 0.6 μg/ml trypsin (Sigma, St. Louis, MO, USA). Plates were incubated for 48 hours (37 C, 5% CO2) and then the agarose removed, cells fixed (70% ethanol) and stained with 1% crystal violet. Virus plaques were counted and the plaque forming units per ml (pfu/ml) were determined using the formula: (# plaques × dilution factor)/0.1 ml. All samples were run in duplicate.

### Multiplex and ELISA for detection of cytokines

Lung homogenate supernatants were assayed for a panel of cytokines and chemokines (IL-1 α, IL-1β, IFN-γ, TNF-α, IL-12 (p70), IL-6, KC, MIP-1β, RANTES and MCP-1) using a multiplex-assay (Bio-Rad, Hercules, CA, USA) according to the manufacturer's instructions. Briefly, 50 μl of a 1× anti-cytokine bead dilution were added to activate 96-well filter plates (100 μl of Bio-Plex Assay Buffer A) and then the plates were washed (2× - 100 μl of Bio-Plex Wash Buffer A). Lung cell suspensions were gently thawed, centrifuged (1 min at 12,000 rpm) and added to the appropriate wells (50 μl). The plates were then incubated with shaking (300 rpm) in the dark for 30 min at 25°C, followed by washes (3×) and addition of the detection antibodies (25 μl/well). Plates were incubated in the dark (30 min, shaking at 300 rpm), followed by three washes, and then 50 μl of a 1:100 dilution of Streptavidin-phycerythrin (PE) was added. Following the 10 min incubation, the plates were washed (3×) and then the beads in each well were resuspended in 125 μl of Bio-Plex Assay Buffer A. The plates were shaken for 30 sec (1,100 rpm) and immediately read using a Luminex^® ^200™ Total System machine (Luminex Corp, Austin, TX, USA). A total of 100 beads per region in a sample volume of 50 μl were counted. The data were analyzed using the LDS1.7 Software. All samples were assayed in duplicate.

Additional cytokines (IFN-α, IFN-β and TGF-α) were assayed by sandwich ELISA (PBL Biomedical Laboratories, Piscataway, NJ and eBioscience, San Diego, CA, USA). Briefly, 100 μl of lung homogenate supernatants were added to anti-cytokine coated plates and incubated at RT for 1 hr. Plates were washed (200 μl) with the appropriate wash buffer, followed by the addition of the appropriate detecting anti-cytokine biotinylated antibody (100 μl). Plates were incubated for 24 hr (IFN-α) or 1 hr (IFN-β and TGF-α) at RT. Following 3 washes with the appropriate buffers, avidin-HRP reagent (100 μl) were added and the plates were incubated for 1 hr at RT. Finally, 100 μl of TMB substrate was added to each well and the plates were incubated in the dark for 20-30 min. The reaction was stopped with 50 μl sulfuric acid (2N) and the colorimetric analysis was determined by a spectrophotometer at an optical density of 450. Cytokine concentrations were determined by linear regressions using the standard curve provided in the kit as reference. Each sample was assayed at least in duplicate.

### Flow cytometry

Isolated lung cells (1-2 × 10^6^) were stained for flow cytometry (FACS) analysis. Briefly, cells were resuspended (1 × 10^7^/ml) in FACS buffer (1× PBS, 3% FBS, 1% Sodium Azide, 1 mM EDTA). Cells (100 μl) were plated in v-shaped 96-well plates and gently centrifuged (1200 rpm, 3 min, 4°C) to pellet. Supernatants were removed and then cells were blocked with mouse anti-Fc antibody (BD Pharmigen, San Jose, CA) for 20 min. Cells were stained with the appropriate antibody panels specific for cell surface markers conjugated to specific flurochromes, CD19-APC-Cy7, CD69-PE-Cy7, CD8-FITC, CD11b-PE, and CD11c-PE-Cy7, (BD Pharmigen, San Jose, CA, USA) or CD3-APC-Cy7, CD4-APC, CD69-PE, CD49b-Pe-Cy7, CD40-APC, MCH Class II-Alexa Fluor 700, F4/80-APC and Ly-6G-FITC (eBioscience, San Diego, CA, USA), as well as their isotype controls. Cells were then washed (2×) with 200 μl of FACS buffer and stained for viability (Live/Dead fixable blue-fluorescent reactive dye, Molecular probes, Eugene, OR, USA) for 30 min. Samples were washed PBS (2×) and then FACS buffer (1×). Finally, cells were fixed with 1% formalin and stored at 4°C. Samples were collected in a BD LSR II flow cytometer and the data were analyzed by FlowJo software (Tree Start Inc. Ashland, OR, USA).

Influenza specific CD8+ cells were assayed using HA_518-526 _(IYSTVASSL) and NP_147-155 _(TYQRTRALV) MHC class I (H-2Kd) restricted immunodominant peptides sequences conjugated to Pentamers-PE (ProImmune, Oxford, UK).

For intracellular staining 2 × 10^6 ^cells were plated and stimulated with HA (IYSTVASSL) and NP (TYQRTRALV) MHC class I (H-2Kd) restricted peptides (1 ug/ml) (Pepscan, Lelystad, The Netherlands) for 5 hours (37 C, 5% CO2) in media containing Monensin (eBioscience, San Diego, CA, USA) and Brefeldin A (Sigma, St Louis, MO, USA). Following stimulation, the cells were surface stained as described before and then permeabilized and stained with intracellular antibodies: IFN-gamma-PE-Cy7 (BD Pharmigen, San Jose, CA, USA), TNF-alpha-FITC (eBioscience, San Diego, CA, USA) and IL-2-PE (eBioscience, San Diego, CA, USA).

### Myeloperoxidase (MPO)

MPO was assayed by ELISA (HyCult, Uden, The Netherlands) in lung supernatants of infected mice (three per time point). Harvested lungs were harvested and forced into suspension in 3 ml of lysis buffer (200 mM NaCl, 5 mM EDTA, 10 mM tris, 10% glycine, 1 mM PMSF), 1 ug/ml leupeptide and 28 ug/ml aprotinine, at pH 7.4). A 1:16 dilution in 1× PBS was assayed following the suggestion of the manufacturer.

### Hemagglutination inhibition (HAI) Assay

HAI assay was used to assess functional antibodies to the HA able to inhibit agglutination of turkey red blood cells (TRBC). This assay was performed as described previously [13]. Briefly, one part sera were treated with three parts of receptor destroying enzyme (RDE) (37°C - overnight). RDE was later inactivated by incubation at 56°C (30 min) and six parts of PBS added. RDE-treated sera were two-fold serially diluted in v-bottom microtiter plates. An equal volume of PR8 virus, adjusted to approximately 8 HAU/50 ml, was added to each well. The plates were covered and incubated at room temperature for 20 min followed by the addition of 1% TRBC (Lampire Biologicals, Pipersville, PA, USA) in PBS. The plates were mixed by agitation, covered, and the RBCs were allowed to settle for 30 min at room temperature. The HAI titer was determined by the reciprocal dilution of the last well which contained non-agglutinated RBC. Positive and negative serum controls were included for each plate.

### Statistics

Differences in weight loss, sickness score, virus titer, cytokine/chemokines and flow cytometry data between mice of the same age group were analyzed by one-way ANOVA, followed by Dunnett's post test. Analysis of results from aged and adult mice at different time points (multiparametric) was assessed by two-way ANOVA tests. Significant was identified as p < 0.05, the data were further analyzed by a Bonferroni post-test. Significant differences in survival were measured using log-rank and Wilcoxon-Gehan tests. Statistical analyses were done using GraphPad software.

## Results

### Enhanced morbidity in aged mice following infection with influenza virus

**Figure 1:**
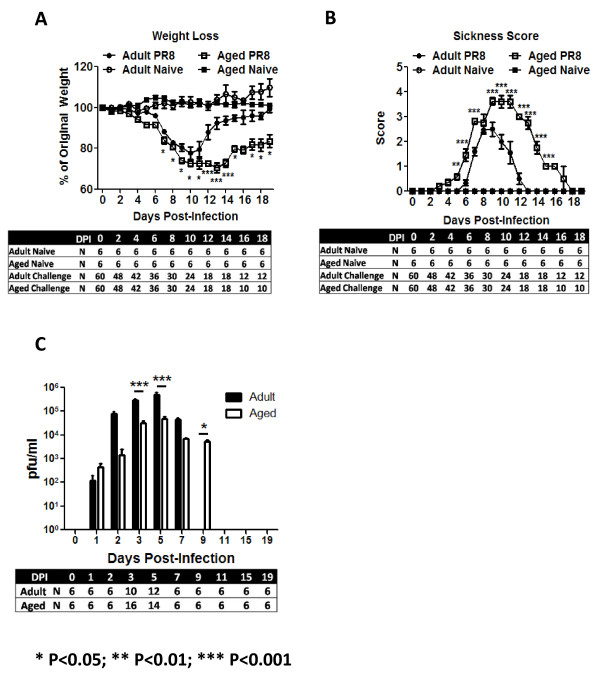
**Mouse Morbidity and Lung Virus Titer**. Following infection with a sublethal dose of PR8 virus, mice were evaluated daily for weight loss (A) and increase in sickness score (B). Adult mice initial weight was significantly lower than aged animals (21.27 ± 0.2785 vs 24.93 ± 0.5157; P < 0.01). Aged animals (white squares) lost weight quicker, had a higher sickness score and recovered slower than adult mice (black circles). Mock infected animals (white circles -- adults; black squares - aged) did not lost weight or showed signs of disease. Influenza virus titer was evaluated in lung supernatants of adult (black bars) and aged (white bars) mice by plaquing on MDCK cells (C). Aged and adult mice had high virus titer in the lungs. Virus was recovered for a longer period of time in aged animals (C), which correlated with the delayed weight recovery (A) from aged animals and delayed reduction in sickness score (B). This suggested alterations in the immune system of elderly animals. Panels A and B are composites of three different experiments and panel C of 2 experiments. In each graph, the arithmetic mean ± SEM are displayed. The number of animals (N) used to generate each graph is displayed at the bottom of each panel. Stars indicate statistical difference between aged and naïve animals. *P < 0.05. **P < 0.01. ***P < 0.001.

In order to develop a comprehensive understanding of the immunological reaction to influenza infection by the aged immune system, lungs from aged (72-76 weeks-old) and adult (12-16 weeks-old) mice were collected following a sublethal dose of influenza PR8 virus (50-100 pfu). Following infection, both adult and aged mice had a rapid drop in body weight until day 9 post-infection (Fig. 1A). Adult mice rapidly recovered and returned close to their original body weight by day 15 post-infection. This recovery in body weight corresponded to a drop in morbidity as defined by sickness score (Fig. 1B). In contrast, aged mice had a slower recovery, a prolonged period of morbidity, and did not return to pre-infection body weight by day 19 post-infection. Despite higher viral lung titers in adult mice (Fig. 1C), all adult and almost all aged mice survived infection (100% vs 96.6%). Furthermore, viruses were recovered for two extra days in aged animals (Fig. 1C).

**Figure 2:**
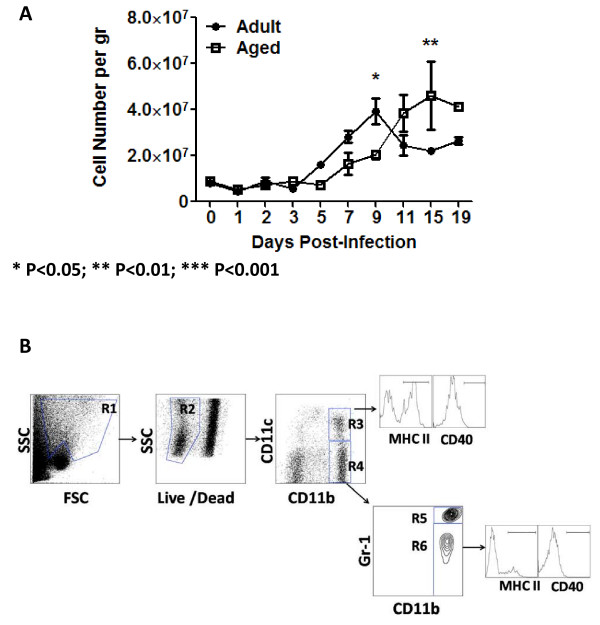
**Lung Cell Infiltration**. Isolated lung cells were evaluated for viability by trypan blue exclusion (A). The number of cells was adjusted for the weight of the lungs and reported as cells per gram of tissue. Aged mice (white squares) showed a delay in the kinetics of lung cell. The graph shows cumulative data of three sets of experiments, a total of 9 mice were assayed at each time point, except for days 15 and 19, which contain 8 animals each. (B) Gating strategy used to analyze innate cells in the lungs. Initially cells were gated in the non-lymphocyte area (R1), this was followed by selection in the live gate (R2). Two APC populations (R3 and R4 gates) were selected based on CD11b and CD11c expression. R3 gate corresponds to cDCs (CD11c^high^/CD11b^high^). Cells in R4 gate were further divided based on the diffential expression of Gr-1 (neutrophils (CD11b^high^/CD11c^low^/Gr1^high^) and lung macrophages (CD11b^high^/CD11c^low^/Gr1^med^). MHC class II and CD40 expression was analyzed in R3 and R6 gates. An average of 500,000 events was collected for the analysis of these populations. Stars indicate statistical difference between aged and naïve animals.*P < 0.05. **P < 0.01. ***P < 0.001.

The total number of cells in the lung began to increase in adult mice at day 5 post-infection and peaked at 4 × 10^7 ^cells/gram on day 9 (Fig. 2). In aged mice, cells began to increase in the lungs at day 7 post-infection. The total cell number peaked at day 15 (~4.5 × 10^7 ^cells/gram) in aged mice; 4 days later than in adult mice (Fig. 2).

### Enhanced number of innate cells in the lungs of influenza infected mice

**Figure 3:**
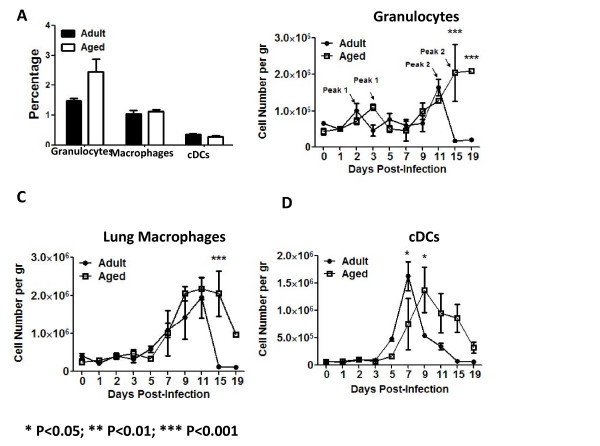
**Innate Cells in the Lungs**. Naïve mice (day 0) had no statistically significant differences in the innate populations analyzed by flow cytometry (A); however, aged animals had the tendency to have a higher percentage of granulocytes. There was a delay in the kinetics of granulocyte and cDCs infiltration in the lungs of aged mice (white squares) (B and D), which suggested alterations in chemokine production. Lung macrophages did not show altered infiltration kinetics, but cleared more quickly from the lungs of adult mice (black circles) (C), which correlated with a quicker recovery from adult mice. Stars indicate statistical difference between aged and naïve animals.*P < 0.05. **P < 0.01. ***P < 0.001.

Cells infiltrating the lung were phenotyped to identify different populations of innate and adaptive cells post-infection (Fig. 2B and Table 2). Among the innate cells, granulocytes (CD11b^high^/CD11c^low^/Gr1^high^), conventional dendritic cells (cDCs) (CD11b^high^/CD11c^high^) and macrophages (CD11b^high^/CD11c^low^/Gr1^med^) were assayed. Gating and characterization of these cell populations in the lung has previously been reported [14,15]. Granulocytes, cDCs and macrophages correspond to R5, R3 and R6 gates on Fig. 2B. The last two populations represent antigen presenting cells (APCs) [16,17]. Expression of F4/80 was low on all these populations (data not shown). Prior to viral infection, there was not a statistical difference in the percentage of lung cells staining positive for these populations in aged mice compared to adult mice (Fig. 3A). However, there was a trend for a higher percentage of granulocytes in aged mice. Following infection, there was a delay in the number of granulocytes and cDCs cells, but not macrophages, infiltrating into the lungs of aged mice compared to adult mice (Fig 3C-D). cDCs peaked by day 7 in adults and day 9 in aged mice (Fig. 3D). Finally, granulocytes had a biphasic increase in cell numbers in the lungs. Initially in adult mice, cells spiked on day 2 and again on day 11 (Fig. 3B). A similar delayed pattern was detected in aged mice with the highest spikes in granulocytes by days 3 and 15 post-infection.

**Table 2:** Adaptive Immune Cell Markers

Markers	B Cells	Activated B Cells	CD4+ T Cells	Activated CD4+ T Cells	CD8+ T Cells	Activated CD8+ T Cells	NK Cells
CD19	Positive	Positive					

CD3	Negative	Negative	Positive	Positive	Positive	Positive	Negative

CD4			Positive	Positive	Negative	Negative	

CD8			Negative	Negative	Positive	Positive	

DX5	Negative						Positive

CD69		Positive		Positive		Positive	

Isolated lung cells were immunophenotyped using various antibody panels. This table summarizes the basic definitions used for each population studied in the adaptive compartment.

### Activation markers of innate cells

**Figure 4:**
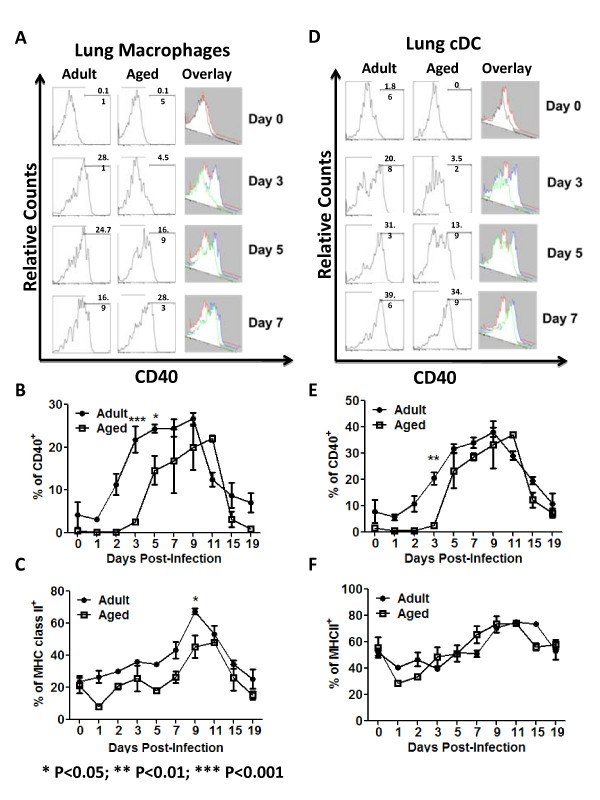
**Innate Cells Activation**. The expression of CD40 and MHC class II was assayed in lung macrophages and cDCs (R6 and R3 in Fig 2B, respectively). Example of expression of CD40 by these cells is shown in adult (first column) and aged (second column) animals at different time points (A and D). An overlay of the histograms is shown to compare differences in up-regulation (third column) of the marker. Panels B and C show upergulation of CD40 and MHC class II respectively. Panels B and C display the average of two different experiments, on which of 3 mice were assayed at each time point (a total of 6 animals per time point), bars represent SEM. Aged animals are represented in white squares and adult animals in black circles. Expression of CD40 on lung macrophages and cDCs was similar in adult and aged animals at day 0. Up-regulation of this marker was slower in aged animals and statistically significant differences were detected as early as day 3 (B and E). On the other hand, MHC class II expression at day 0 and up-regulation was similar between adult and aged animals in both lung macrophages and cDCs (C and F). For expression of activation markers an average of 500,000 events were collected. *P < 0.05. **P < 0.01. ***P < 0.001.

Activation of APCs was determined by the up-regulation of CD40 and MHC class II cell surface markers. The surface expression levels of CD40 on lung macrophages and cDCs collected from adult and aged mice was similar prior to infection (Day 0) (Fig. 4A and 4D). Cell surface CD40 on lung macrophages was significantly up-regulated within 2-3 days post-infection in adult mice, but did not increase on the same cells from aged mice until day 5 post-infection (Figs. 4B). Similar CD40 up-regulation patterns on lung cDCs from adult mice were detected (Figs 4E). MHC class II expression was also similar on lung macrophages and cDCs of adult and aged animals at day 0. Despite that lung macrophages from adult mice appeared to up-regulate MHC class II more quickly than the same cells from aged animals, the differences were not statistically significant, except for day 9. Similarly, no differences were detected between lung cDCs from adult and aged animals (Fig. 4D). For activation of granulocytes, the production of myeloperoxidase (MPO) was assessed. Both adult and aged mice produced similar levels of MPO, except for day 9 post-infection, in which aged mice had a significantly higher level of MPO (data not shown).

### Cytokines and chemokines produced by macrophages and cDCs

**Figure 5:**
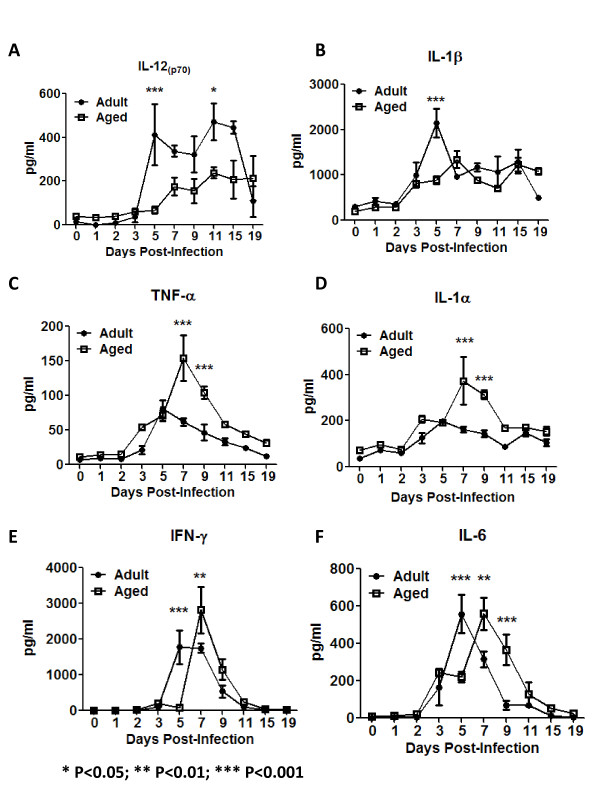
**Pro-inflamatory and lymphocytic cytokines**. The concentration of several pro-inflamatory and lymphocytic cytokines was determined in the supernatants of lung homogenates. IL-12_p70 _(NK and CD4 T-cell stimulant) was produced earlier and at significantly higher levels in adult mice (black circles) (A), which correlated with an earlier detection of IFN-γ in adult animals. IL-1β concentrations peaked at day 5 post-infection in adult mice (black circle), but steadily increased in aged mice (B). TNF-α and IL-1α concentrations increased similarly in both adult and aged mice until day 5 and then declined over the 19 days of observation in adult animals (black circles)(C and D). In contrast, these two cytokines continued to increase in aged mice (white squares) with significantly higher concentrations at day 7-9 post-infection (C and D). The lymphocytic cytokines, IFN-γ and IL-6, spiked earlier in adult mice (black circles) (E and F). The graphs represent cumulative results of two different experiments. The arithmetic mean (± SEM) of the cytokine(s) concentration of 4-6 mice assayed at each time point was plotted. Stars indicate statistical difference between aged and naïve animals.*P < 0.05. **P < 0.01. ***P < 0.001.

**Figure 6:**
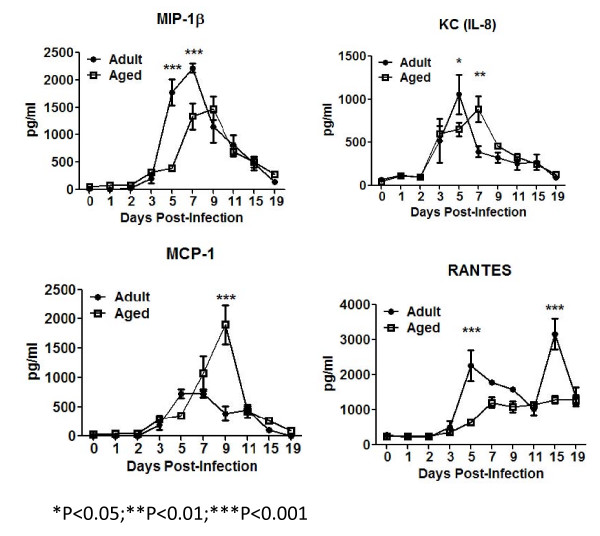
**Chemokines**. Several chemokines were determined in the lung supernatants of aged and adult mice infected with influenza virus. MIP-1β increased and peaked earlier in adult animals (black squares) (A). KC increased similarly in the lungs of adult (black circles) and aged (white squares) mice, however peaked earlier in adult animals (B). MCP-1 had a mild early peak in adult mice (black circles), while a higher delayed peak in aged animals (white squares) (C). There was a biphasic expression pattern of RANTES in adult mice (black circles) with a peak in concentrations at day 5 and 15 (D), while aged animals (white squares) had a very mild increase in this chemokine. The overall delayed production of chemokines correlated with delays in infiltration kinetics of innate and adaptive cells the lungs. The graphs represent cumulative results of two different experiments. The arithmetic mean (± SEM) concentration of 4-6 mice assayed at each time point was plotted. Stars indicate statistical difference between aged and naïve animals. *P < 0.05. **P < 0.01. ***P < 0.001.

Activated immune cells produce various cytokine and chemokines following influenza infection [18-20]. In order to determine if the delay in activation of aged immune cells may be a result of impairment cytokine secretion, supernatants of lung homogenates were assessed for a panel of cytokines/chemokines (Fig. 5, 6). IL-12_p70 _(active form of IL-12) was detected at higher concentrations in the lungs of adult mice as early as day 5 post-infection (~400 pg/ml) compared to aged mice, which had a slower rise and plateau at half the concentration (~200 pg/ml) (Fig. 5A). Adult mice maintained an elevated level of IL-12_p70 _between days 5 and 15 post-infection before declining. In adult mice, IL-1β (pro-inflammatory cytokine) concentrations peaked at day 5 post-infection, but steadily increased in aged mice (Fig. 5B). Interestingly, TNF-α and IL-1α (pro-inflammatory cytokines) concentrations increased similarly in both adult and aged mice until day 5, when the levels of these two cytokines declined over the 19 days of observation in adult animals (Fig. 5C and 5D). In contrast, these two cytokines continued to increase in aged mice with significantly higher concentrations at day 7-9 post-infection (Fig. 5C and 5D). This might reflect the increased tendency to produce inflammatory mediators that has been described in elderly individuals [21,22].

The concentrations of IFN-γ and IL-6, both highly produced by lymphocytes, began increasing in the lungs of adult mice at day 5 post-infection and 2-3 days later in aged mice. Despite of the earlier increase of IFN-γ in adult animals, the peak of this cytokine was higher in aged animal (day 7 p < 0.01) (Fig. 5E). On the other hand, the peak of IL-6 was similar in both ages of mice (Fig. 5F). There were no differences in the levels of IFN-α and IFN-β cytokines measured in the supernatants of lung homogenates between adult and aged mice (data not shown).

MIP-1β and KC (murine homologue of human functional IL-8 [23]) are important chemokines for attraction of lymphocytes and granulocytes to the site of infection, respectively [23,24]. The concentration of these chemokines, increased in the lungs of adult mice 2-3 days post-infection, peaking at days 5-7 (Fig. 6A and 6B). In aged animals, the rise of MIP-1β was delayed by 2 days and peak production of MIP-1β and KC were delayed by 2 days compared to adult mice (Fig. 6A and 6B). Both chemokines returned to baseline levels by day 19 post-infection. MCP-1, a monocyte chemoattractant, was elevated in the lung of aged mice (Fig. 6C), which followed a similar pattern as TNF-α and IL-1α. There was a biphasic expression pattern of RANTES (monocyte and lymphocyte chemoattractant [25,26]) in the adult mice with a peak in concentration at day 5 and then again at day 15 post-infection (Fig. 6D). In aged mice, there was an increase in RANTES between days 3-5 that was 2-4 fold lower than observed in adult mice, which then plateau at ~1500 pg/ml for the remainder of the experiment.

### Lymphocytic immune cell phenotypes following infection

**Figure 7:**
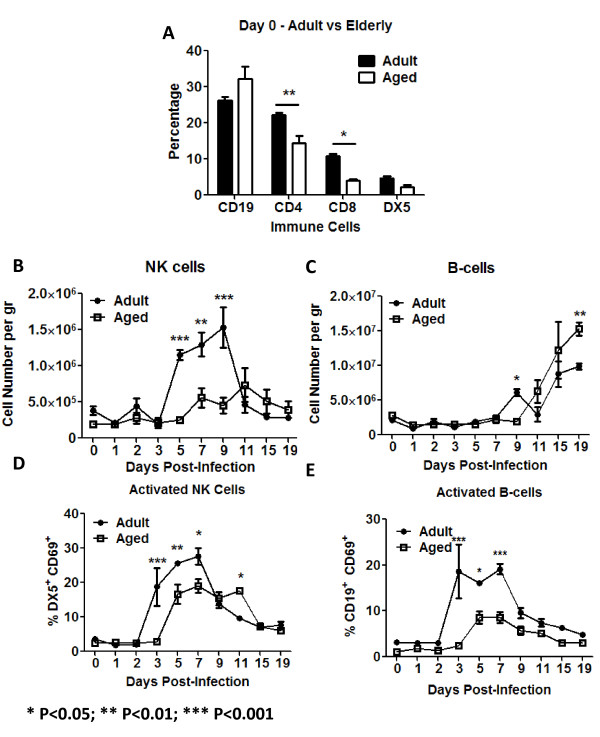
**Lymphocytic cells**. Several innate and adaptive lymphocytic cells were analyzed in the lungs of aged and adult mice. CD4^+ ^and CD8^+ ^T-cells were more abundant in adult mice (black bars) at day 0 (A). Adult mice (black circles) had a significantly higher infiltration of NK (DX5) cells in the lungs (B). The kinetics of B-cell lung infiltration, on adult (black circles) and aged (white squares) mice were similar (C). Expression of the early activation marker CD69 by NK and B-cells was detected earlier and at a higher percentage in adult mice in (black circles) (E and F), which suggested that despite similar infiltration kinetics, adult B-cells started to produce antibodies earlier. This was confirmed later when HAI were performed (Fig. 8). The graphs represent cumulative results of two different experiments. The arithmetic means (± SEM) of the number of cells (C and D) or percentages (A, D and E) at each time point (4-6 mice per time point) were plotted. Stars indicate statistical difference between aged and naïve animals. *P < 0.05. **P < 0.01. ***P < 0.001.

**Figure 8:**
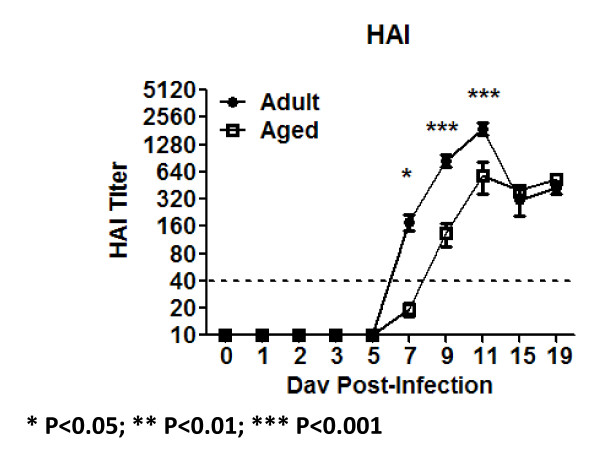
**Anti-Influenza Neutralizing Antibodies**. Anti-influenza neutralizing antibodies titers were assayed in sera samples by HAI assay. Aged animals (black circles) developed antibodies at neutralizing titer (1:40 -- dotted line) quicker than aged mice (white squares). The arithmetic mean (± SEM) of the sera HAI titer at each time point was plotted. Six mice per time point were assayed, except at days 7 and 15, where 10 mice were assayed. Stars indicate statistical difference between aged and naïve animals. *P < 0.05. **P < 0.01. ***P < 0.001.

There were no significant differences in the baseline levels of B cell (CD19^+ ^CD3^- ^DX5^-^) or natural killer (NK) cells (DX5^+ ^CD3^- ^CD19^-^) between aged and adult mice (Fig. 7A). NK cells in the lungs of adult mice increased 3 fold between 3 and 5 days post-infection and peaked at day 9 (1.5 × 10^6 ^cell/gram), whereas there was no significant increase in the number of NK cells in the lungs of aged mice (Fig. 7B). B cells in both aged and adult mice began to increase in the lungs at day 11 post-infection, with slightly higher number of B cells in the lungs of aged mice (Fig. 7C). There was a delay in the number of activated cells in these two cell populations in the lungs of aged mice compared to adult mice, as indicated by surface expression of CD69, with fewer percentages of activated cells in aged than adult mice (Fig. 7D and 7E). Consistent with a delayed activation of B-cells in aged animals, hemagglutination-inhibition (HAI) specific antibodies were detected later in aged mice than in adult mice (Fig. 8).

### Influenza specific T-cell responses

**Figure 9:**
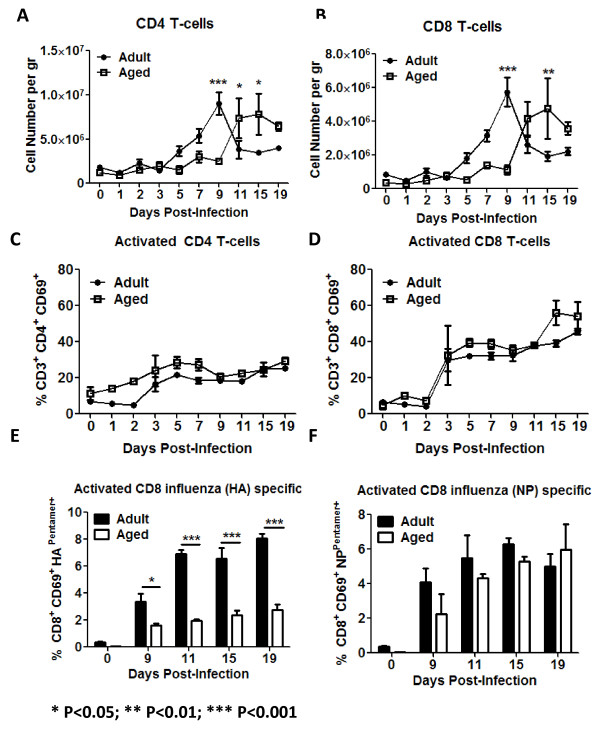
**T-lymphocytes**. Correlating with a delayed detection of cytokines and chemokines in the supernatants of lung homogenates (Figs. 5 and 6), CD4^+ ^and CD8^+ ^T-cell infiltration was delayed in the lungs of aged animals (white squares) (A and B). However, up-regulation of CD69 was similar in aged and adult animals (C and D), which suggested non-specific up-regulation of this marker by these cells. Confirming this, activated influenza specific cells were detected only after day 9 post-infection (E and F). Plots A to D represent cumulative results of two different experiments. The arithmetic means (± SEM) of the number of cells (A and B) or percentages (C and D) at each time point (4-6 mice per time point) were plotted. In panels E and F, 5 mice per time point were assayed bars represent the arithmetic mean (± SEM). Stars indicate statistical difference between aged and adult animals. *P < 0.05. **P < 0.01. ***P < 0.001.

**Figure 10:**
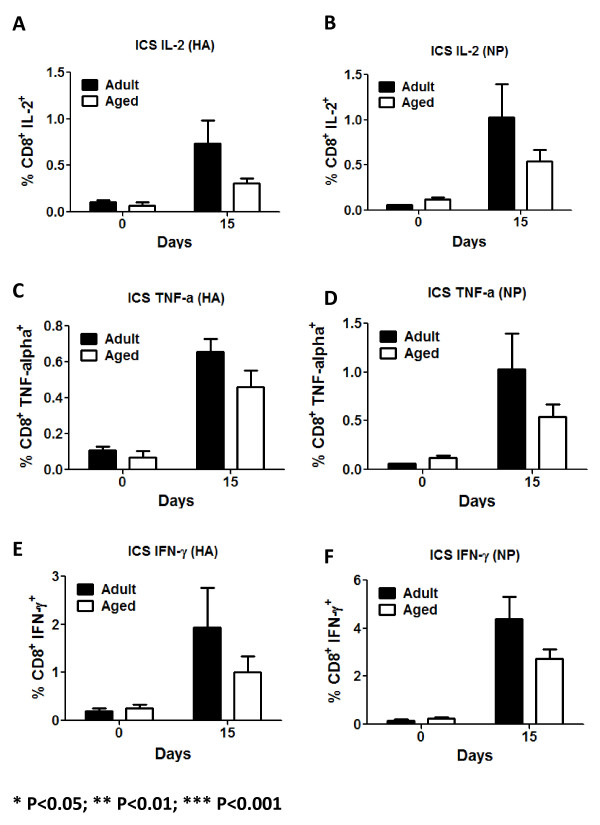
**Intracellular Cytokine Staining**. IL-2 (A and B), TNF-α (C and D) and IFN-γ (E and F) production were assayed in CD8^+ ^T-cells following stimulation with immunodominant influenza peptides at day 15. Even though not statistically significant differences between adult and aged animals were detected, adult animals had the tendency to have a higher percentage of cells producing these cytokines regardless of HA or NP stimualtion. Five to eight mice per time point were assayed. Bars represent the arithmetic mean (± SEM). Stars indicate statistical difference between aged and naïve animals. *P < 0.05. **P < 0.01. ***P < 0.001.

Adult mice had a significantly higher baseline percentage of lung CD8^+ ^(CD3^+ ^CD8^+^) and CD4+ (CD3^+ ^CD4^+^) T-lymphocytes compared to aged mice (Fig. 7A). However, there was a delay in the infiltration of new CD4^+ ^and CD8^+ ^T-cells in aged mice compared adult mice (Fig. 9A and 9B). Both subpopulation of T-cells increased in the lungs of adult mice at day 5 and peaked at day 9 (Fig. 9A and 9B). These same T-cell populations did not start increasing in the lungs of aged mice until day 11 post-infection. Despite differences in overall cell number in the lungs between adult and aged mice, there was no significant difference in the activation of either T-cell population between adult and aged mice (Fig. 9C and 9D). To further test this, immunodominant influenza MHC class I (HA_518-526 _IYSTVASSL and NP_147-155 _TYQRTRALV) restricted epitopes were used. There were significant differences in influenza HA-specific activated CD8^+ ^T-cells between adult and aged mice between days 9 and 19 (Fig. 9E). There were no significant differences in the percentage of activated CD8^+ ^T-cells specific to NP (Fig. 9F). No statistically significant differences in the percentage of HA or NP specific CD8+ T-cells producing IL-2, TNF-α or IFN-γ were detected between adult and aged mice by day 15. Despite this, adult mice had the tendency to have a higher percentage of cytokine producing CD8^+ ^cells (Fig 10A - F).

## Discussion

Influenza virus affects all ages of humans; however, the elderly (>65 years-old) have increased susceptibility to infections and are especially predisposed to complications [6]. The increased morbidity and mortality reported in elderly populations are due to several factors that include subjacent chronic diseases, such as diabetes and cancer, as well as dysfunctions in the senescent immune system. The mouse model has proven to be an important tool to explore the pathogenesis of influenza, especially due to the similarities to human infections [27]. The elderly mouse model has also proven to be useful to explore the effects of age on the immune responses to several respiratory infectious agents including *Mycobacterium tuberculosis *and influenza virus [18,28,29]. However, most of the studies have focused on the T-cell compartment and indicated that altered T-cell proliferation and memory results in decreased and delayed CTL activity [4,28,30-33]. Few reports have addressed the role of the aging innate immune system on the protection to influenza virus and only few reports have explored phagocytic functions, NK cell activity, and IFN-α/β production in the aged animals using other pathogens [34-40]. In this study, the immune responses to influenza virus in the lungs of aged animals were evaluated. Alterations in APC up-regulation of CD40 correlated with delayed production of cytokines and chemokines in the lungs, which correlated with infiltration of immune cells into these organs. Finally, this correlated with a delayed activation of the adaptive immune responses and subsequent delay in clearance of virus.

The earlier weight loss and enhanced morbidity in aged animals suggests less effective innate immune responses against influenza virus in these mice. Among the cells of the innate immune system, granulocytes, DCs and macrophages are critical players, since these are the first cells to encounter a pathogenic microorganism. Among granulocytes, neutrophils are the predominant population (>90%) and the role of these cells in influenza infection has recently been elucidated [39,41]. One mechanism of granulocyte activity is the release of lytic proteins, such as myeloperoxidase, from endocytic granules. Aged animals had delayed lung infiltration kinetics following influenza infection (Fig. 3B); however, similar levels of MPO were produced by adult and aged mice, except for day 9, where higher levels were detected in the aged group (p < 0.05) (data not shown). This indicates that for the main part, the neutrophil function was not altered in aged animals infected with influenza virus. The higher MPO levels at day 9 might contribute to the enhanced sickness score detected in aged animals at this time point. The data also suggest that the difference in infiltration kinetics may be associated with impaired chemotaxis. Consistent with this hypothesis, the secretion of the chemokine, KC (neutrophil chemoattractant), was delayed in aged mice following influenza virus infection (Fig. 9B).

Interestingly, the major differences in granulocyte infiltration kinetics were detected between days 11-19 (Fig. 3B), which corresponds with the resolution phase of the disease. The higher granulocyte infiltration in aged animals also correlated with a prolonged presence of macrophages (Fig. 3C) and was associated with a higher sickness score and prolonged disease. A prolonged infiltration of the lungs with cells from the inflammatory phase might account for the prolonged disease stage in the animals. To further support this, MPO was higher at day 9 in aged animals and reports that aged populations have a higher tendency to produce inflammatory cytokines, such as TNF-α and IL-α, during infections have been published and correlates with our data (Fig. 5C) [21,22]. In summary, all these immune markers suggest that the immune system can be contributing to the enhanced sickness score detected in the aged group of animals.

APCs are among the first leukocytes to recognize infectious microorganisms. DCs are especially responsible of the surveillance in different tissues and subsequent migration to the lymph nodes where they interact with T-cells to present antigens and trigger the adaptive immune response. In the murine lung, different DC populations have been recently described, one of the predominant populations includes resident CD11b^high^/CD11c^high ^cells, also known as conventional DCs (cDCs) (reviewed in [17]). The other APC populations analyzed in these animals represent lung macrophages (CD11b^high^/CD11c^low^/Gr1^high^). It is important to note that lung (interstitial) macrophages are different from alveolar macrophages. The later cells suppress the functional characteristics of lung DCs [17]. Alveolar macrophages in addition to CD11c, express F4/80 [42]. Both APC populations analyzed in our experiments have low F4/80 expression (data not shown), providing evidence that the analyzed cells were not alveolar macrophages. Upon encountering the antigen, APCs up-regulate several molecules, such as MHC class II and CD40 in order to present antigens to CD4^+ ^T-cells and provide the required second signal to fully activate these cells [43]. MHC class II up-regulation was not altered neither in lung macrophages nor cDCs from aged mice following influenza infection. However, CD40 up-regulation was delayed in both lung macrophages and cDCs cells, suggesting differences in complete priming of the APCs in aged mice. Fully primed APCs produce cytokines and chemokines that will attract cells to the lungs and subsequently activate them during influenza virus infection [44,45]. CD40 interaction with CD40L, is important to fully activate APCs [46,47] and the role of CD40 in stimulating the production of IL-12 is well documented (reviewed in [43,48]) The concentration of IL-12_p70_, the active form of IL-12, was not only significantly higher in the lungs of adult mice, but also spiked earlier compared to aged mice following influenza infection (Fig. 7A). Considering the importance of CD40 in the induction of IL-12, the delayed CD40 up-regulation in aged animals, most likely contributed to a retarded IL-12_p70 _production and reduced activation of aged APCs.

In addition to IL-12, activated APCs can produce other pro-inflammatory cytokines, such as IL-1β, IL-1α, and TNF-α (Fig. 5) [19,20,49]. Interestingly, not all of these cytokines showed higher concentrations in adult mice compared to aged mice following influenza infection; however, in all cases the peak of cytokine level occurred earlier in adult animals. The higher peak of the pro-inflammatory cytokines detected in elderly animals correlate with reports indicating that elderly populations have enhanced basal levels of these cytokines (e.g. TNF-α) and tendency to produce higher levels upon infection [21,22,50]. Similar to cytokines, the peaks of chemokine production by APCs (MIP-1β, MCP-1, RANTES and KC) to influenza infection were delayed in aged mice. This correlated with the delayed CD40 up-regulation in aged animals, which further suggests a delay in full activation of APCs.

IL-12_p70 _produced by activated APCs stimulates NK cells and CD4^+ ^T-cells to produce IFN-γ [48,51,52]. Consistent with the delayed production of IL-12_p70 _in the lungs of aged animals to influenza infection, IFN-γ spiked two days later (day 7 vs. day 5) in aged mice compared to adults (Fig. 5E). Consistent with the delayed production of the chemokines, NK cells, CD4^+ ^and CD8^+ ^T-cells showed delayed infiltration into the lungs of aged animals (Figs. 7B, 9A and 9B). Furthermore, the up-regulation of the early activation marker CD69 was delayed on NK cells in aged mice (Fig. 7D). Previous reports had shown no alterations of NK cells with age in humans and only small changes in mice [53-57]. However, our data coincides with a recent report that demonstrates alterations in the NK compartment of aged animals infected with influenza virus [40].

B-cells showed similar patterns of infiltration between aged and adult mice following influenza infection (Fig. 7C). The kinetics of this population were delayed compared to CD4^+ ^and CD8^+ ^T-cells (Fig. 9), since B-cells started to infiltrate the lungs between days 9 and 11, upon which these cells significantly increased in adult and aged animals (Fig. 8D). Despite this, surface expression of CD69 was detected by day 3 in adults and day 5 in aged mice (Fig. 7E), which coincided with an earlier detection of anti-influenza neutralizing antibodies in adult mice compared to aged mice (Fig. 8). Interestingly, CD69 up-regulation by CD4+ T-cells was similar between adult and aged mice (Fig. 9C and 9D), suggesting that the differences in IFN-γ production detected between days 3 to 9 between aged and adult mice is primarily due to secretion of this cytokine by NK cells.

There was a delay in the infiltration of CD4^+ ^and CD8^+ ^T-cells into the lungs of aged mice compared to adult mice (Fig. 9A and 9B), however, there was little difference in the activation of these cells (Fig. 9C and 9D). Early CD69 up-regulation by T- lymphocytes in the lungs is dependent on IFNs type I production [58,59]. Consistent with this, no differences in IFN-α/β production in the lung supernatants of aged or adult animals were detected. Early CD69 up-regulation by T-cells most likely represented non-specific activation of these cells, which may have inefficiently produced IFN-γ. Furthermore, recent reports suggest that the lung airway environment might also play an important role in the up-regulation and maintenance of CD69 by lymphocytes [60]. In a primary infection, influenza specific cells, might play a more prominent role than nonspecifically activated cells. To determine this, influenza specific activated (CD69^+^) CD8^+ ^T-cells were assayed using HA and NP immunodominant epitopes [61,62] conjugated to pentamers of MHC class I molecules. Consistent with our hypothesis that early up-regulation of CD69 by T-cells was not influenza-specific, activated (CD69^+^) CD8^+ ^influenza specific T-cells were detected only after day 9 post-infection. Furthermore, the percentage of HA_518-526 _specific activated CD8^+ ^T-cells were consistently higher in adult mice compared to aged mice (Fig. 9E). In contrast, the percentage of NP_147-155 _specific activated CD8^+ ^T-cells were not statistically different between aged and adult animals (Fig. 9F); nevertheless, adult animal had a higher percentage of CD8^+^/CD69^+ ^NP-pentamer^+ ^T-cells between days 9 and 11 post-infection (Fig. 9F). Considering the delay in virus clearance in aged mice (Fig. 1C), HA specific CD8^+ ^T-cells most likely play a prime role in virus clearance and the late appearance of these cells in aged mice most likely contributed to the prolonged recovery. Despite that no statistically significant differences were noted by day 15, adult mice showed the tendency to have a higher percentage IL-2, TNF-α and IFN-γ producing CD8^+^T-cells regardless of HA or NP stimulation (Fig 10). Since mature (fully primed) DCs efficiently induce cytokine production by CD8^+ ^T-cells and the generation of cytotoxic T cells [63], the reduced cytokine production by aged CD8^+ ^T-cells might be the result of reduced APC activation as suggested by alteration in CD40 up-regulation. This could eventually lead to a delayed virus clearance in the lungs.

Virus titration in the lungs of aged animals showed a delayed virus clearance. However, the data also demonstrated that despite a similar onset of virus between adult and aged animals, the peak was lower in the later set of animals. This might be the result of a reduced homeostasis of epithelial cells of aged animals. Aging reduces the division potential of cells (replicative senescence) both *in vivo *and *in vitro*. Some of these changes have been only partially explored in the lungs; however, affect most organs in the models used [64-67]. A reduced replication capability can be translated in reduced virus production by aged animals during peak days. Even with lower virus titer in the lungs of aged animals, the alterations in the immune responses (innate and adaptive) might account for reduced virus clearance and the enhanced tendency to produce inflammatory cytokines (e.g. TNF-α) [21,22] by these animals might be responsible for the enhanced sickness score.

The data in this study shows that age affects the global immune responses to influenza infection. Alterations in CD40 up-regulation by aged cDCs and lung macrophages suggested impairments in their activation. Remarkably, this correlated with altered levels of cytokines (especially IL-12_p70_) and chemokines, which also correlated with delayed NK and T-cell infiltration. Furthermore, influenza (HA)-specific T-cells were reduced in aged animals. These findings correlated with several reports demonstrating that age affects APC Toll-like receptors expression and function, antigen presentation (defect in exogenous pathway) and CD8+ stimulating capacity [50,68-71]. Some studies, on the other hand, have not reported defects in DC function in the elderly [72]. This might indicate that different populations of APCs at different tissues are affected differently by age. Also, this may be the result of genetic differences between mouse strains. Our data also demonstrated altered humoral immune responses (B-cell activation and HAI titers). Therefore, the alterations in the APCs are most likely just one step in a large chain of alterations present in the aging immune system. The final outcome of delayed virus clearance and slow recovery is probably the addition of various factors and not only involve dysfunction in antigen presentation.

## Conclusion

The current study provides an overview of the global effects that aging has on the immune system following influenza infections and demonstrates that alterations are found in the innate, as well as acquired immune compartment. Remarkably, alterations in lung APCs seem to trigger a cascade of events that affected the acquired immune responses. According to the US Census Bureau [73], during 20^th ^century, the rate of growth of the elderly population has greatly exceeded the growth rate of the US population as a whole. The elderly increased by a factor of 11, from 3 million in 1900 to 33 million in 1994, while the total population and the population younger than 65 years of age tripled. It is expected that by 2030 the elderly population will double. Considering the significant increase in the elderly population and increased susceptibility to certain infectious agents, it is becoming imperative to determine the effects of age on the immune system. Identifying the main groups of cell(s) affected by the aging process may help to further explore the mechanisms behind the immune alterations and design better vaccines and adjuvants to boost the immune responses in this important group of the population.

## Competing interests

The authors declare that they have no competing interests.

## Authors' contributions

FRT carried out the experimental work, the data analysis, and drafted the manuscript. TMR and FRT participated in the design of the study. TMR and FRT conceived the hypotheses, advised on experimental work and assisted in drafting the manuscript.
